# Detection of Coronary Artery Disease by an Erectile Dysfunction Questionnaire

**DOI:** 10.1155/2021/6647995

**Published:** 2021-03-13

**Authors:** Mehrab Sayadi, Reza Elmafshar, Iman Razeghian-Jahromi, Mohammad Javad Zibaeenezhad

**Affiliations:** Cardiovascular Research Center, Shiraz University of Medical Sciences, Shiraz, Iran

## Abstract

**Background:**

Erectile dysfunction (ED) has been become an important health challenge in recent years affecting the quality of life significantly. In addition to imposed social problems, it may warn the existence of cardiovascular diseases especially that of ischemic heart disease (IHD). We aimed to investigate the association between ED and coronary artery disease (CAD) in a population of patients with stable angina based on angiographic findings.

**Methods:**

In this cross-sectional study, among patients who are diagnosed with stable angina referring for coronary angiography (excluding those with acute coronary syndrome), 200 patients were selected. They were divided equally into two groups of case and control. The former were positively CAD patients and the latter were normal peers, with respect to angiographic results. International index of erectile function (IIEF) questionnaire was used in order to evaluate erectile function during recent four weeks. Statistical analyses of the *t*-test and logistic regression were performed. The significance level was considered as a *P* value less than 5%.

**Results:**

The age range of the patients was 40–65 years old. The case group was significantly older (*P*=0.001). There was a remarkable relation between the low score from IIEF (ED) and existence of CAD. Also, the severity of ED was in a close relationship with severity of CAD. In addition, dyslipidemia in terms of high LDL and low HDL was associated with both ED and severity of CAD.

**Conclusion:**

Other than CAD, ED could be considered as one of the manifestations of atherosclerosis. Accordingly, the IIEF questionnaire is a useful tool to early diagnosis of CAD. Also, IIEF-derived scores estimate CAD severity. We suggest subjects with low score of IIEF examine their cardiovascular health with special attention to possible existence of IHD.

## 1. Background

Erectile dysfunction (ED) is known as persistent disability to keep erection during a sexual intercourse [1]. ED is a common clinical manifestation that affects mainly men of more than 40 years of age. Underlying reasons of ED include, but not limited to, diabetes mellitus, hypertension, obesity, insufficient physical activity, and lower urinary tract diseases [2]. The association between ED and cardiovascular disease (CVD) has been documented previously. ED is a strong predictor for coronary artery disease (CAD). Cardiovascular assessment of a noncardiac patient presenting with ED is a useful recommendation [3]. Existence of cardiovascular risk factors augments the correlation between ED and CAD. Penile vascular disorder in men with ED is associated with significant increase in established cardiovascular risk factors such as fasting lipids, fasting sugar, body mass index, C-reactive protein, and homocysteine [4, 5]. Reduced peak penile systolic velocity is correlated with the risk of CAD as well as with the degree and distribution of atherosclerotic lesions [6]. Men with ED generally show more severe CAD and left ventricular dysfunction than peers without ED. The severity of ED is also in relation with the severity of CAD [6, 7].

Erectile-related problems are associated with elevated mortality rate of CVD [4, 5]. ED may predict the presence of CAD in the absence of cardiac symptoms as confirmed by the multidetector computed tomography system that identifies subclinical plaques [8]. Clinical studies revealed that diagnosis of ED in healthy men as well as in patients with type II diabetes may be related to the subclinical CAD that is not detectable with stress testing [9]. It was assumed that erectile abnormality possibly is a probable marker of early cardiovascular events, even before the plaque rupture [10]. In the current study, we are going to evaluate the ED relationship with CAD and its severity in patients with stable angina based on angiographic findings.

## 2. Methods

This cross-sectional study was designed in conformity with the Helsinki declaration and has approved by the vice-chancellor of research and technology of Shiraz University of Medical Sciences. We selected patients among those who referred to defined clinics during 2018-2019. Participants were asked to sign an informed consent. All the participants were diagnosed with stable angina pectoris. After routine examinations such as taking history and noninvasive tests such as echocardiography, the exercise tolerance test, and heart scan, they were referred for coronary angiography based on cardiologist decision. Other inclusion criteria were male gender with age between 40 and 65 years old. Those with acute coronary syndrome, the history of diabetes mellitus, the history of rheumatologic diseases, urologic disorders (chronic urinary tract infection and sexual diseases because of urological problems), hormonal disorders, previous percutaneous coronary intervention, the history of coronary artery bypass grafting, congenital anomalies, inflammatory bowel disease, psychological diseases, and patients on relevant medication for more than one month were excluded, all based on validated documents. According to the results of coronary angiography, patients were divided into two groups. The case group included 100 patients with CAD which defines 50% or more intraluminal narrowing in one or more native coronary arteries or their branches. The control group included 100 patients with normal coronary arteries based on angiography reports which mean less than 50% narrowing in previously described arteries. We measured triglycerides (TG), high-density lipoprotein (HDL), and low-density lipoprotein (LDL) in the participants as well.

### 2.1. ED Evaluation

International Index of Erectile Function (IIEF) criterion was used in this regard [11]. This 15-item questionnaire is a valid self-assessment tool that has been found useful in the clinical evaluation of ED. According to the history of sexual activity in the past four weeks, a score from 0 to 5 is given to each question. The total score is calculated by summing up of all 15 items. So, the maximum score would be 75, with the lower score indicating worse erectile function.

### 2.2. Statistical Analysis

Data were presented as mean ± SD and number (%) for continues and categorical variables, respectively. The *t* test, adjusted unconditional logistic regression, and receiver operator characteristic (ROC) were used as appropriate in the SPSS for Windows, version 16.0, Chicago, SPSS Inc. *P* value <0.05 was considered as the significant level.

## 3. Results

All the patients were 40–65 years old with the mean of 61.08 ± 4.28. There was a statistical difference in age between two groups (*P*=0.001). The IIEF score was also significantly different (Table 1). Regarding lipid profile, unlike TG, the case group showed significantly higher LDL and lower HDL levels compared with the control group (*P* < 0.001) (Table 1). Correlation between TG and the IIEF score was not significant (*r* = −0.11, *P*=0.100). While IIEF showed significant reverse correlation with LDL (*r* = −0.370, *P* < 0.001), its correlation with HDL was direct (*r* = 0.270, *P* < 0.001).

**Figure 1:**
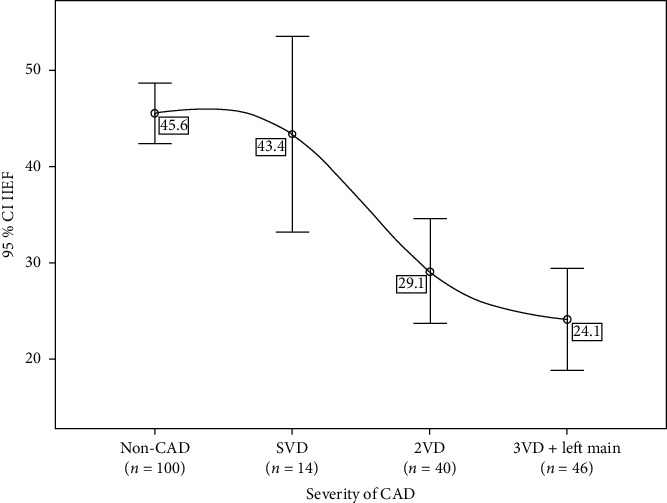
Mean score of IIEF in the non-CAD group and 3 CAD groups. CAD, coronary artery diseases; SVD, single-vessel disease; 2VD, two-vessel disease; 3VD, three-vessel disease. We indicate severity of CAD according to number of involved vessels. Figure shows that as CAD becomes more severe, mean of IIHF decreases, and severity of ED increases.

Multiple unconditional logistic regression showed the relationship between low scores of IIEF and CAD (Table 1).  Figure 1 demonstrates that severity of CAD in terms of number of diseased vessels was accompanied with the low IIEF score or ED severity.

**Figure 2:**
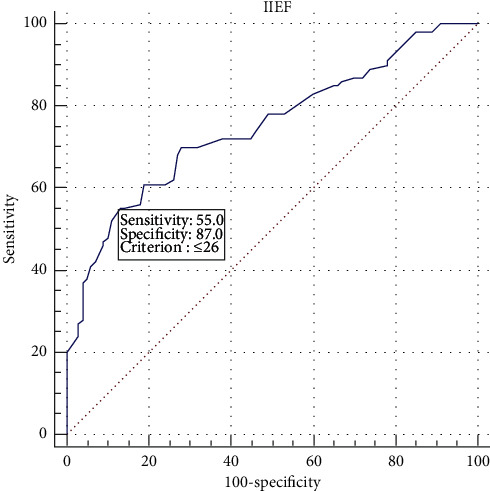
ROC curve for the IIEF score. The optimal cutoff point for IIEF was 26. Area under the ROC curve (AUC) was 0.750 with 95% CI (0.648–0.808) and *P* < 0.001. The sensitivity and specificity of IIEF as a screening test was 55% and 87%, respectively. Note: area under the curve indicates the predictive power. Predictive power will be high if it is far from 0.5 value (close to 1 value).

ROC curves were used to determine the predictability of the IIEF score (Figure 2). According to area under the curve, the IIEF score discriminates the case from the control group with sensitivity of 55% and specificity of 87%. The area under the ROC curve was 0.750 with 95% CI (0.648–0.808). It became 0.690 with 95% CI (0.58–0.79) after adjustment for age, LDL, and HDL as confounding covariates. These results indicate that the ED can predict CAD after excluding the confounding factors.

## 4. Discussion

The association between ED and CVD has been reported in different previous studies. Patients with CVD are at high risk for experiencing sexual dysfunctions [12, 13]. The results of the present study indicated that the IIEF score was significantly different in the case versus the control group.

In a study, the prevalence of ED patients with and without CAD was reported to be statistically different. ED patients with CAD had significantly higher levels of uric acid and lipoproteins than peers without CAD [14]. In this report, LDL and IIEF scores were mentioned to be independent predictors of CAD. Men who suffer preexisting ED are prone to develop more severe CAD than those without ED. In a 15-year follow-up interval, ED was shown as a significant predictor of all-cause death and the composite of cardiovascular death, myocardial infarction, stroke, and heart failure in men with CVD [14].

The risk of developing a cardiac event within a 10-year time duration is raised by approximately 1.5 times in men with ED in comparison to men without ED [15]. Males with ED had significantly higher incidence of atherosclerotic events [16]. Preexisting ED was in relation to increased risk of peripheral vascular disease. Frequency of ED was reported to be 48.6% in type II diabetic patients with CAD and 39.7% in peers without CAD. The data confirmed that ED was a reliable predictor of silent CAD in younger diabetics [17]. Common risk factors such as obesity, smoking, diabetes, hypertension, and hyperlipidemia were recognized in both ED and CAD facilitating their incidence [18, 19].

In the present study, lipid profile including TG, LDL, and HDL were measured. Differences of HDL and LDL between the case and control groups were statistically significant, but the TG level did not show any difference between two groups. Hyperlipidemia was reported as a usual finding in ED patients, similar to our study.

Hyperlipidemic patients possess a high risk of being involved by CAD [20]. Further reports have implied that high lipoprotein-a (Lp-a) in CAD patients is correlated with ED [15]. Also, the high level of lipoprotein-associated phospholipase A2 (Lp-PLA2) is a predictor of ED, and concurrently, Lp-PLA2 plays a prominent role in the formation of oxidized LDL which is a crucial player in initiation and development of CAD [9, 21].

CAD does not always lead to myocardial ischemia [22]. It means IHD may occur in the absence of atherosclerotic plaques [23]. In healthy condition, epicardial coronary arteries involve a bit in coronary vascular resistance [24]. These are the arterioles that play a critical role in microcirculation and constitute more than half of the coronary resistance [25]. Pathophysiological conditions such as arterial hypertension, dyslipidemia, diabetes mellitus, and genetic variations cause chaos in the controlling of vascular resistance. Meanwhile, failure in adaptation of coronary blood flow to myocardial metabolic demands lead to coronary microvascular dysfunction (CMD) [22]. Coronary blood flow is dysregulated due to some reasons including impairment in coronary ion channels, increased shear stress, and escalated exposure of LDL, ROS, inflammation mediators, and advanced glycation endproducts to endothelial surfaces [22].

Clinical symptoms of IHD were observed in patients with CMD in the absence of CAD [26]. Interestingly, mortality rate of patients with CAD is as same as peers with CMD without CAD [26–28]. Both CAD and CMD could be independent causes of myocardial ischemia. However, they may occur concurrently in such patients [27]. Reasonably, penis with considerable need of blood supply during sexual intercourse is influenced by microvascular dysfunction as well. However, there are no imaging tools for visualization of coronary microcirculation, and the real contribution of coronary microcirculation to IHD [24] or ED pathophysiology is not clearly understood.

It has been supposed that different manifestations of ED and CAD are related to the differences in the size of the arteries that supply the penis and the myocardium [10]. Atherosclerosis is a multivessel disorder, and nearly, all vessels can theoretically be damaged with the similar pathology. A dramatic reduction in blood flow may not appear in larger arteries (myocardium) as much as smaller ones (penis) at the same degree of atherosclerotic plaque formation.

It does not still confirm whether men with ED show overt symptoms of CAD, whereas those with CAD will often complain about preexisting ED symptoms. Recent findings revealed that the existence of ED is related to more severe cardiovascular events as nonsymptomatic atherosclerosis is initiated and developed several years before the appearance of its symptoms [29].

Early signs of CAD are detected in a significant proportion of men with ED. Some researchers have found that the time interval between the beginning of ED symptoms and the occurrence of CAD symptoms is as few as several years. It was reported for more than 3 years until cardiac infarction, after initiation of ED symptoms [30], though longer time intervals have been reported as well [31]. ED is associated with a significant increase in CAD in younger patients, but it is less associated with CAD in older individuals [32]. In younger patients (less than 70 years), CAD is anticipated following ED, while in men older than 70 years, there is uncertainty about the prognostic value of ED [31].

The risk of acute coronary syndrome (ACS) is evident in many of subjects with ED [33]. Extensive involvement of coronary arteries in terms of left main and three-vessel disease was reported in ACS patients who suffered from moderate or severe ED [34]. ED predicts poor outcome such as rehospitalization and death in patients with acute MI [35]. However, ED appears less in patients with ACS, while it is more prevalent in the case of chronic coronary syndrome [36].

Asymmetrical dimethylarginine (ADMA) is an inhibitor of nitric oxide synthase indicating endothelial dysfunction and cardiovascular abnormality [33]. It is significantly overexpressed in ED and coronary atherosclerosis cases, even those in the early stages [37]. Also, ADMA level is correlated to the occurrence of ACS [38]. However, neither ED severity nor the ADMA level was not appropriate predictors of major cardiovascular events in patients with ACS [33].

Men who suffer erectile abnormality should accomplish a thorough medical assessment, with respect to blood pressure, fasting lipids, and glycaemia status in order to risk stratification of cardiovascular events and applying early medical intervention. Medical treatment for risk factors including diabetes mellitus, hypertension, hyperlipidemia, and smoking cessation should be regarded as useful strategies to lower the risk of ED and CAD. Weight loss besides sufficient physical exercise possibly improves erectile function.

This study suffers from inherent limitations of cross-sectional studies. Also, the participants were selected from a single center, and all of them are from the same ethnicity. All the participants had stable angina, and patients with other cardiovascular disorders were not considered in this study. Small sample size restricts the generalizability of this study. Findings of our study would be examined in large multicenter prospective investigations. Self-assessment of erectile function may lead to bias because of personal desire to underestimation or overestimation of the reality.

## 5. Conclusion

Atherosclerosis affects all the arteries with similar pathology. In our population of stable angina, ED could be considered as an early indicator of CAD. Indeed, severity of CAD is in relation with scores of the IIEF questionnaire.

We suggest subjects with low score of IIEF examine their cardiovascular health with special attention to possible existence of IHD.

## Figures and Tables

**Table 1:** Association between IIEF and CAD without and with adjusting other variables.

Variables	CAD^+^ (*n* = 100)	CAD^−^ (*n* = 100)	*P* value	Adjusted OR	95% CI for OR		*P* value
					Lower	Upper	
Age	62.08 ± 2.85	60.08 ± 5.17	0.001	1.128	1.028	1.237	0.011
TG	158.4 ± 46.13	151.02 ± 52.0	0.289	0.997	0.990	1.004	0.433
LDL	152.07 ± 45.8	113.56 ± 42.03	<0.001	1.012	1.004	1.020	0.002
HDL	39.9 ± 3.96	44.08 ± 5.15	<0.001	0.862	0.796	0.933	<0.001
IIEF	28.81 ± 18.32	45.58 ± 15.88	<0.001	0.958	0.938	0.978	<0.001

CAD, coronary artery disease; OR, odds ratio; CI, confidence interval; TG, triglycerides; LDL, low-density lipoprotein; HDL, high-density lipoprotein. Data were presented as mean ± standard deviation (SD). Analyses were performed using the *t* test and multiple unconditional logistic regression for unadjusting and adjusting covariates. There is a relation between ED and CAD by controlling the other covariates.

## Data Availability

The datasets analyzed during the current study are available from the corresponding author upon request.

## Abbreviations

ED: Erectile dysfunction
IHD: Ischemic heart disease
CAD: Coronary artery disease
IIEF: International Index of Erectile Function
TG: Triglyceride
HDL: High-density lipoprotein
LDL: Low-density lipoprotein.
